# O Exercício de Caminhada Melhora a Variabilidade da Pressão Arterial Ambulatorial em Claudicantes

**DOI:** 10.36660/abc.20210140

**Published:** 2021-05-06

**Affiliations:** 1 Universidade Federal do Rio Grande do Sul Porto AlegreRS Brasil Universidade Federal do Rio Grande do Sul - Programa de Pós-Graduação em Ciências da Saúde: Cardiologia e Ciências Cardiovasculares, Porto Alegre, RS - Brasil; 2 Hospital de Clínicas de Porto Alegre Porto AlegreRS Brasil Hospital de Clínicas de Porto Alegre - Grupo de Pesquisa em Cardiologia do Exercício, Porto Alegre, RS - Brasil; 3 Hospital de Clínicas de Porto Alegre Porto AlegreRS Brasil Hospital de Clínicas de Porto Alegre - Grupo de Vascular e Exercício - VascuEx, Porto Alegre, RS - Brasil

**Keywords:** Doença Arterial Periférica, Prevalência, Índice Tornozelo – Braço, Pressão Arterial, Aterosclerose, Diabetes Mellitus, Claudicação Intermitente, Caminhada

A doença arterial periférica (DAP) tem se mostrado cada vez mais prevalente em todo o mundo^1^. O diagnóstico clínico se baseia na avaliação do índice tornozelo-braquial (ITB), onde a pressão arterial (PA) sistólica do tornozelo é dividida pela PA sistólica do braço². Valores <0,9 indicam a presença de DAP. A redução da PA sistólica do tornozelo se deve à aterosclerose dos membros inferiores. Indivíduos com DAP apresentam alterações hemodinâmicas significativas, como aumento nos níveis de PA^3^. Além disso, a alta variabilidade da PA está correlacionada ao desenvolvimento de DAP em indivíduos diabéticos^4^.

É importante reduzir os níveis de PA em indivíduos com DAP. O treinamento físico é uma ferramenta útil que pode auxiliar no tratamento dos sintomas clínicos^5^^,^^6^. Foi o que Chehuen et al., fizeram; eles investigaram o efeito do exercício de caminhada (EC) na variabilidade da PA ambulatorial em indivíduos com DAP. Trata-se de um elegante ensaio clínico randomizado no qual indivíduos com DAP e sintomas de claudicação foram divididos em dois grupos: controle (n=16) e EC (n=19). Avaliou-se a PA ambulatorial de 24 horas antes e após 12 semanas. Como desfecho, eles avaliaram a PA sistólica média e a PA diastólica ambulatorial, bem como as variáveis que representam a variabilidade da PA sistólica, diastólica e média (desvio padrão de 24 horas — DP24; desvio padrão ponderado em vigília e sono — DPdn e média de 24 horas de variabilidade real — MVR24).

Como principal resultado, o grupo EC conseguiu reduzir as variabilidades de PA sistólica e média em comparação com o grupo controle. Um estudo simples, mas extremamente relevante, que mostra que o EC é eficaz para melhorar a variabilidade da PA ambulatorial em indivíduos com DAP. A seguir encontram-se alguns pontos interessantes do artigo. O grupo controle realizou 30 minutos de alongamento duas vezes por semana. Este é um detalhe importante para um ensaio clínico randomizado atual. É necessário que se disponibilize tempo de intervenção semelhante com a mesma frequência semanal para os dois grupos. O EC consistiu de 15 minutos de caminhada na esteira seguido de um intervalo de 2 minutos (30 minutos de exercício ativo e 30 minutos de descanso). A intensidade foi controlada pela frequência cardíaca referente ao limiar de claudicação (padrão ouro para prescrição de DAP), com velocidade padrão de 3,2 km/h e ajuste de gradação quando necessário^8^.

O desenho do estudo também mostra a alta qualidade metodológica com a correta alocação dos participantes^9^. A realização de ensaios clínicos randomizados no Brasil é difícil, devido ao alto custo e escassez de mão de obra. É difícil realizar cegamento em estudos com exercícios físicos, uma vez que caminhada é bem diferente de alongamento. Porém, para efeito de comparação, um grupo controle é obrigatório^10^. Os autores optaram pelo alongamento, mas poderiam ter disponibilizado palestras sobre educação postural, atividade física e estilo de vida, por exemplo. O mais importante é que todos os avaliadores de resultados estavam cegos para o tipo de intervenção, garantindo a confidencialidade da alocação^11^.

O estudo tem muitos méritos. Porém, é importante notar que alguns pontos relacionados às pequenas limitações devem ser destacados. Um deles é que o EC é realizado em esteira, o que reduz a validade externa em nível populacional, uma vez que muitas pessoas não possuem esteira e caminham ao ar livre. Poderia haver mais um grupo que fizesse a caminhada na rua, para comparar os efeitos com a esteira, por exemplo. Além disso, é altamente recomendável que indivíduos com DAP realizem treinamento de resistência para melhorar os níveis de resistência muscular e até mesmo o perfil lipídico. Portanto, eles poderiam ter traçado uma comparação entre as diferentes modalidades de treinamento nesses indivíduos no contexto da PA. Todos esses comentários podem ser usados como incentivo para novos estudos.

Por fim, a DAP é uma doença subdiagnosticada e, por muitos anos, as pessoas apresentam sintomas sem um diagnóstico definitivo. A melhora nos níveis de variabilidade da PA pode ter um impacto favorável na redução do risco cardiovascular e na melhora do prognóstico da doença. Portanto, a criação de estratégias de incentivo e engajamento em programas de exercícios físicos é extremamente necessária para essa população.
