# Supplementary material for: O Exercício de Caminhada Melhora a Variabilidade da Pressão Arterial Ambulatorial em Claudicantes
Source: Arq Bras Cardiol. 2021 May 6;116(5):906–7. [Article in Portuguese] doi: 10.36660/abc.20210140 (PMC8121462; doi:10.36660/abc.20210140)
Supplement: Translation

# Translation: Walking Training Improve Ambulatorial Blood Pressure Variability in Claudicants

**Affiliations:** 1 Porto AlegreRS Brazil Universidade Federal do Rio Grande do Sul - Programa de Pós-Graduação em Ciências da Saúde: Cardiologia e Ciências Cardiovasculares, Porto Alegre, RS - Brazil; 2 Porto AlegreRS Brazil Hospital de Clínicas de Porto Alegre - Grupo de Pesquisa em Cardiologia do Exercício, Porto Alegre, RS - Brazil; 3 Porto AlegreRS Brazil Hospital de Clínicas de Porto Alegre - Grupo de Vascular e Exercício - VascuEx, Porto Alegre, RS - Brazil

**Keywords:** Peripheral Arterial Disease, Prevalence, Ankle Brachial Index, Arterial Pressure, Atherosclerosis, Diabetes Melllitus, Intermittent Clauduication, Walking

Peripheral arterial disease (PAD) has been shown to be increasingly prevalent worldwide.^1^ Clinical diagnosis is based on evaluation of the ankle brachial index (ABI), where the ankle systolic blood pressure (BP) is divided by the systolic BP of the arm.² Value <0.9 indicate the presence of PAD. Reduction in systolic BP of the ankle is due to atherosclerosis in the lower limbs. Individuals with PAD have significant hemodynamic abnormalities, such as an increase in BP levels.^3^ In addition, high BP variability is correlated with the development of PAD in diabetic individuals.^4^

It is important to reduce BP levels in PAD individuals. Physical training is a useful tool that can assist the treatment of clinical symptoms.^5^^,^^6^ It was what *Chehuen* et al.^7^ did; they investigated the effect of walking training (WT) on ambulatory BP variability in PAD individuals. It is an elegant randomized clinical trial in which individuals with PAD and claudication symptoms were divided into two groups: control (n=16) and WT (n=19). 24-hour ambulatory BP was assessed before and after 12 weeks. As an outcome, they evaluated the mean systolic BP and ambulatory diastolic BP, as well as variables representing systolic, diastolic and average BP variability (24-hour standard deviation – SD24, awake and asleep weighted standard deviation – SDdn, and 24-hour average real variability – ARV24).

As a main result, the WT group was able to reduce systolic and mean BP variabilities compared to the control group. A simple but extremely relevant study, showing that WT is effective to improve the variability of ambulatory BP in PAD individuals. Following are some interesting points of the manuscript. The control group performed 30 minutes of stretching twice a week. This is a key detail for a current randomized controlled trial. It is necessary that a similar time of intervention be made available with the same weekly frequency for both groups. WT included 15 minutes of walking on the treadmill followed by a 2-minute interval (30 minutes of active exercise and 30 minutes of rest). The intensity was controlled by heart rate referring to the claudication threshold (gold standard for prescription of PAD), with a standard speed of 3.2 km/h and grade adjustment when necessary.^8^

The study design also shows the high methodological quality with the correct allocation of the participants.^9^ Conducting randomized clinical trials in Brazil is difficult, due to the high cost and shortage of labor. It is difficult to perform blinding in studies with physical exercise, since walking is quite different from stretching. However, for comparison purposes, a control group is mandatory.^10^ The authors opted for stretching, but it could be lectures on postural education, physical activity and lifestyle, for example. Most importantly, all outcome assessors were blinded to the intervention type, ensuring allocation confidentiality.^11^

The study has many merits. However, it is important to note that some points related to small limitations should be highlighted. One is that the WT is performed on a treadmill, which reduces the external validity at the population level, since many people do not have a treadmill and walk outdoors. There could be one more group that would walk on the streets to compare the effects with the treadmill, for example. In addition, it is highly recommended that individuals with PAD undergo strength training to improve their muscle strength levels and even their lipid profile. Therefore, they could have made a comparison between different training modalities in these individuals within the context of BP. All of these comments can be used as an incentive for further studies.

Finally, PAD is an underdiagnosed disease, where, for many years, people have had symptoms without a closed diagnosis. Improving BP variability levels can have a favorable impact on the reduction of cardiovascular risk, and improve disease prognosis. Therefore, creating incentive and engagement strategies in physical exercise programs are extremely necessary for this population.
